# Supplementary material for: No Women’s Land: Australian Women Veterans’ Experiences of the Culture of Military Service and Transition
Source: Int J Environ Res Public Health. 2024 Apr 15;21(4):479. doi: 10.3390/ijerph21040479 (PMC11050049; doi:10.3390/ijerph21040479)
Supplement: Supplementary file 1 [file ijerph-21-00479-s001.zip › ijerph-2867338-supplementary.pdf]

Supplementary File: 3.4.2 Adjustment, disconnection and the invisible veteran

This file contains for subtheme detail, including descriptive information and supporting direct quotes from participants (pseudonym, age, years of service)

**_1. Initial adjustment_**<br>For some participants, there was difficulty in learning new life skills, given the young age at which they had joined the military and the way in which the military had taken care of their needs for food, shelter and healthcare.	
Separation meant not only adjusting to loss of routine but to loss of the immediate visibility of a military rank and status in the civilian world.<br><br>Others commented on how military training and acculturation becomes ingrained in military behaviour which is difficult to undo and is misaligned with civilian life.	*...Kept on working because I always had done, except that I wasn't wearing a uniform and people didn't call me ma'am anymore. I remember going to the doctors and someone said Mrs X. I didn't do anything...I was never called Mrs X, I was either X or ma'am. (Linda)*<br><br>*I didn't know what a Medicare card was, and how to use it, and what it was for, because straight from school, straight in that, and everything's free. You need an appointment; you just go somewhere...And you feel so stupid...So coming out I was like, 'oh well I don't have skills that the realworld needs'...it's like cooking. I can't cook, because I never had to. (Pauline, mid-50s, 20 years)*<br><br>*Yeah, a bit scary really because you are so protected in the military…I have always been told what time to get up…Now I am on my own…I don't have anybody telling me what to do every five seconds. (Quinn, mid-50s, 20 years)*<br><br>*I've also found myself triggered by military things, which doesn't surprise me. Like, for example, we were in the office the other day, and I don't know where it was, but a whistle went off...a whistle for the flags that go up. It just kind of symbolises got to come to attention, basically and I found my whole body tensed and I was just like, whoa, and I just didn't expect that. (Rochelle, early 30s, 5+ years)*<br><br>*I kept getting pulled up because I swore like a trouper which is perfectly acceptable in a Defence environment, but not so much in the [state]Health environment, and so I realised I had obviously acculturated myself and dropping F bombs and C bombs actually isn't normal but in Defence it is. (Heather, 50 years, 30+ years)*

**2. *Disconnection***<br>There was a sense of disconnection or disengagement with the civilian world in the narratives. For some, this was long standing, the trauma of abuse and the nature of their separation leaving them unable to emotionally connect with others and form meaningful relationships for years, often decades. Leaving the military with loss of purpose and direction, shame and stigma, loss of confidence and military identity pervaded their lives and family relationships. Military rejection had long-term consequences for their sense of self.	
The impacts of poor separation processes on feelings of stigma, guilt, shame, failure and betrayal were also revealed in descriptions by participants.	*I'm wondering, why are my parents not talking to me about what's happened? I must be such a disgrace if they don't even want to know me…I thought I was so abhorrent...I was traumatised. No counselling, no nothing...in trauma because I had no idea what I was meant to be doing, how I was meant to be reacting, what was normal what wasn't normal. I was just drunk all the time...then the mental health side of things…Well, I lost my son, I lost my marriage, I lost my job, I lost my home, I lost everything. I was homeless for the first six months of this year... I was in a DV relationship for 14 years. (Sarah, 50 years, <1 year)*<br><br>*The Army never physically killed me. It emotionally killed me. I'd lost my career and my future...So, I married the first man who said he loved me. He told me he loved me within a week, and I didn't know so I just believed it. End up being a perpetrator of abuse, domestic violence. He was in the military...he had child safety trying to get my kids off me, all this stuff because I was having PTSD. I didn't know what was happening to me. I didn't even know what PTSD was or anything. (Naomi, 50 years, 5 years)*
For many participants, a sense of disconnection from the civilian world was expressed in their choice of friendships. Descriptions revealed the strength of connections made during service and the understanding that these peer connections can provide:	*I avoided everything... it was probably incredibly stressful for my family because I moved back home and they had no idea what had happened really [physical and psychological abuse]. So, it was like a slow process of wanting to talk about things; not knowing what to say; me not knowing what was happening; not having words for what was happening…it was 2018 when I signed off. (Madison, late 20s, <2 years)*<br><br>*I think you develop these really deep friendships and then sometimes you come out and you have, you know, superficial friendships and potentially nothing will be, nothing will ever relate to the experience of being in war. So, in that regard, I think that's the same with men or women. (Chrissie, mid-40s, >10 years)*<br><br>*I still have a very good friend who's a veteran, who's retired now, but she's got four kids and you know, we're good, we look after each other in that respect. But I*

	wouldn't tell anyone else that I had these problems because they wouldn't understand. (Frances, early 70s, 25 years)
3. Invisibility to others<br>Other narratives also revealed experiences of invisibility within the civilian and veteran communities. Participants described the various ways in which their service is either seen as secondary to that of men or is misconstrued.	
Belinda commented on her experience of women in the military being stereotyped as aggressive and authoritarian.	*The male…with military it's kind of like oh you've got these leadership skills. If you're female military, you're too assertive and nobody wants you. And it's the same in a lot of male dominated workplaces. Women, assertive women are not looked upon highly and definitely that was the presumption. (Belinda, late 30s, 5+ years)*
Others commented on their experiences of lack of understanding of the roles undertaken by women in the military. Julie described a sense of invisibility with civilian males until she discloses the nature of her role in the military. She also commented that men have difficulty when she does because her military role was associated with traditional masculine traits of strength, courage and heroism rather a role traditionally associated with women such as support or nursing.	*I find it frustrating when I don't get treated either with respect…when people all of a sudden change their tune when they find out that I am a veteran...I have a skillset that's quite unique...It's kind of like you need to prove yourself first...if I meet with let's say a bunch of businessmen…I have to prove myself before I get any traction. (Julie, early 40s, 20+ years)*
Rochelle and Terri also commented on lack of understanding of the nature of their military roles and the impacted that this has on their veteran identity:	*I think why people in the military don't identify themselves is more just that civilians just don't get it... And they'll be like, oh what, you had a gun? And I'll be like, well yeah, I was in the Army, of course I fire a gun, and yeah...when I say what, like I was in admin in the Army, that people then just assume that maybe I was like, the civvies, like the APS are...that's why I just often prefer not to just say anything. (Rochelle, early 30s, 5+ years)*<br><br>*Me I sat at a desk. The things I heard and the things I was privy to would put me there on the frontline... I don't tell everybody because they look at you weirdly enough as it is. If it comes up, I don't offer that information, if it comes up do you know what I mean? Yeah was in the Army? What did you do? Computer stuff. (Terri, late 50s, <5 years)*
Others, who experienced military sexual trauma, described a need to protect themselves from questions, misunderstandings and potential further distress:	*Oh, I keep it [military service] to myself, yeah. Like I said, it opens too many questions, and I don't have too many good things to say, you know. (Sarah, 50 years, <1 year)*
Katrina described misconceptions about PTS and the invisibility of the service experiences of women for the general public.	*If I identify that I have PTSD then people sometimes furrow their brow and say, "Well hang on, you were a female in the navy with PTSD, how does that work?" Like you weren't on the frontline and it's a case of 'yeah sunshine, but that doesn't mean I didn't see some really nasty shit rescuing asylum seekers that had, had the backs of their legs*

	bitten open by sharks or that had been chopped up by boat propellers or dead bodies in the water, morgue reports…there's all this other baggage that is never even considered because if you're not in the special forces you couldn't have PTSD. (Katrina, early 40s, 10+ years)
Several participants revealed their invisibility as women veterans within the veteran community, together with a lack of recognition of gender-specific needs. Katrina's description revealed experiences of a prevailing misogyny among older men veterans towards women who served:	*Even today on Anzac Day, the old crusties, especially army guys, they'll look at [name of husband] and walk up and start a conversation with him and completely ignore me...the old crusties with the RSL go up and they'll talk to [name of husband] and they'll be going "Oh what's this medal, what's this, what's this?" And my other two girlfriends, they've got heaps of gongs as well and the old guys will just totally ignore them. (Katrina, early 40s, 10+ years)*
This downplaying or affording less value to women's service by men veterans was revealed in some experiences with veteran advocates in claiming compensation from DVA for service injuries. For some, poor attitudes from male advocates only increased the sense of invisibility as veterans.	*I went to see an advocate…the Veteran Review Board...so there was only one available, so I went to see him. Now, he's an old Vietnam vet, and he doesn't know what to do with a woman that's had sexual abuse...it was like, oh, 'I'm an old fuddy-duddy and I don't feel women's stuff'...so when I went into hospital, I told him that this needs to be addressed within 30 days, DVA had given me 30 days to redress it. He said, 'yep, yep, no worries, leave it with me, leave it with me'. So, after about six weeks, I got in contact with him and I said, 'well how are we going with that claim that I needed to' – he goes, 'what claim?'...So, he's overlooked it all, put it on a pile somewhere and forgotten about it. (Eleanor, 50 years, 20+ years)*
4. Invisibility and DVA Twenty-one participants disclosed service-related injuries, the majority of which were for psychological issues, predominantly PTS. Only a few participants described positive interactions with DVA, with the majority of participants describing protracted and difficult claims processes whether for psychological trauma or physical injuries. Again, there is a theme of invisibility of the nature of service experiences for women.	
Invisibility to the support system is revealed in Naomi description of her experiences. The stigma and shame associated with her abuse and the ensuring psychological impacts rendered her service invisible to others for over 20 years. Recent recognition of her veteran status and acknowledgement that her psychological issues were related to service has had a major impact on her life.	*I never knew what DVA was until 2019...it's just overwhelming to sort of get thrown all this fucking money. Bang, bang, we accept liability to your PTSD, we accept liability to your borderline personality...You're telling me I could've had a better life...I have been homeless...Struggling on food stamps and all that. (Naomi, 50 years, 5 years)*
Likewise, Sarah's experiences impacted her life since separation from the military and she stated that, along with her emotional stress, she does not feel worthy or entitled to undertake the claims process with DVA:	*I can't face it if I'm honest...I'm not well enough. I physically couldn't do the work. I suppose I self-excluded in that I don't feel like I served long enough...So I don't even know if I can claim…The other thing that I would love to be able to explore would be to look at a medical discharge, like a retrospective medical discharge and I think that's the*

	pathway that I need to go down now, but I don't even know who to talk to and how to go about it and it's another system and another process. (Sarah, 50 years, <1 year)
5. Health care needs Descriptions also revealed the invisibility of women veterans as mothers and as having gender-specific health and care needs, particularly related to their experiences of MST.	
While most participants no longer had small children, Anna described the invisibility of her needs as a veteran caring for small children in both the DVA and ex-service care systems. She described how her needs for counselling could not be met by Open Arms due to lack of provision of both child-care and breast-feeding facilities and she was advised to come back when her children were older.	*So, it's sort of like I am asking for help now because I need help and you're creating all these barriers for me. It's quite frustrating, and they were like 'oh just go to Open Arms', and it's like Open Arms are causing a lot of problems in their own right. (Anna, early 30s, 10 years)*
Eleanor commented on the invisibility of MST in hospital programs. Having several admissions for suicide attempts and ideation related to her sexual assaults in the military, she highlighted the difficulties she experienced in both being in a hospital environment and being with men.	*I just went in and out of hospital for years. That wasn't conducive to my health, because that's where all the abuse happened, it was in the hospital with doctors and nurses, and psychologists...I've been thrown into situations where I've had to be in hospital with the boys, and it's not a place for a woman to be...it was 10-week exposure program. Oh, my God... I relive my experiences every single day, I don't need to talk about it every single day, and then put on headphones and listen to it every single day, because it doesn't desensitise me to it, it ignites it…and at that point I was drinking a lot of alcohol, and very suicidal. (Eleanor, 50 years, 20+ years)*
Eleanor also commented on issues for women veterans who have experiences of MST but suppress them and their own need for care because of caring for their children.	*I don't feel like there's anything for women that have been through that, and I feel like it's a real taboo, and no one wants to talk about it, and they go, 'oh look, we haven't got any women complaining or whatever'. It's because women get out and they either are single mums or they're mums that are just busy with being a mum and trying to get on with their experiences, and all the feelings that they've suppressed for so many year... And these hospitals don't allow you to express that, because if you do, you get labelled. (Eleanor, 50 years, 20+ years)*
Likewise, Sarah also dealing with long-term impacts of MST commented on the inability of her needs being met in a hospital program which only offered one therapeutic approach.	*I couldn't bear going back to hospital. Due to the idiocy of the way their programming is set up, DVA had a contract with the hospital for a requirement for all DVA patients to do CBT therapies...my psychiatrist said ' It is not helping her. So, no more CBT for her.' And because that contract was there between the hospital and DVA, I had to leave the hospital because I couldn't do the program. So, I am sick but I am not allowed to stay at hospital because I am not well enough to participate in the program that's*

	contracted. (Sarah, 50 years, <1 year)
6. What women veterans want: peer support and safe spaces Participants commented on the types of health care they needed and the difficulties in accessing this care under DVA legislation, revealing an invisibility of the gender-specific healthcare needs of women veterans. They highlighted the need for 'safe' spaces which provide an opportunity for women to come together to talk, connect, and be understood as women veterans.	
Retreats were mentioned as safe spaces by a number of participants.	*There's a farm up in [name of town], it's called Veteran's Retreat, and my friend runs it, and they do women's circles, and they do women's weekends, and they try different things. There's Tai Chi, and there's the Raiki, and all of these alternate things that aren't approved by DVA that actually help. (Eleanor, 50 years, 20+ years)*<br><br>*So, I often go on meditation retreats and I find them really rejuvenating…just the opportunity to be in silence and then you know, the opportunity to talk in safe circles I think is really empowering…provide space and silence as well as some safe spaces to talk you know. (Chrissie, mid-40s, 10+ years)*
The importance of sharing, reflecting and healing was illuminated by a few other participants. In particular, some of the older women described gaining an understanding that their experiences in the military constituted abuse.	*So I look back and I go 'well if that happened now like I absolutely would have reported it'...when new girls joined our unit we would warn them about essentially the predators or the creepy guys in the unit, but no-one ever thought to report them for being predators or creepy guys…I look back and I was like what on earth were we doing? (Heather, 50 years, 30+ years)*<br><br>*My actual time in the service I enjoyed, loved every second…the longer I've been out, the more I reflect, the more resentful I get... it's really only dawned on me probably in the last 4 or 5 years of these things that happened…I just put it down a lot as drunk, and young, and having fun, and not really understanding the whole dynamics of what was going on...the negative things…either I didn't feel them, or I suppressed them. I'm not quite sure. (Pauline, mid-50s, 20 years)*
For others, the MeToo movement and the Royal Commission has provided the impetus to reframe experiences and seek justice:	*This is why I can talk like this. Otherwise, I wouldn't be talking to you. So, I would not – because I would not even know about it...I would've died...Only the MeToo Movement is when I opened my mouth about what happened to me in the army...2018. (Naomi, 50 years, 5 years)*

	When the Royal Commission came about, I was like oh my god that's my chance to voice up…the defence force as a whole really let me down as a young person. I was vulnerable to attack, and I was attacked. (Olga, 50 years, 15+ years)
7. Identity as veterans and connection through advocacy<br>All participants expressed immense pride in having served but revealed diversity in their adoption of a 'veteran' or 'ex-service' identity with many having described how they both protected this identity and how it was dependent on the situation.	
For many participants, being a veteran along with their identities as mother, worker or student required ongoing navigation as a fluid process.<br><br>For several participants, and especially those who experienced military abuse, finding connections with other veterans has helped with restoring self-confidence, esteem and a sense of veteran identity. These participants described engaging in advocacy roles within the veteran community and strongly identify as veterans themselves. For several, a desire to help others stemmed from both seeking justice for military abuse and finding a new sense of purpose. Deirdre described how helping other veterans with their DVA claims processes stems from the lack of care she experienced in the military medical system and enables her to use both her nursing knowledge and lived experience in the military. Advocacy enables her to regain the sense of belonging and camaraderie she had in the military.<br><br>Eleanor described how seeking justice for their experiences of MST has translated into seeking justice for other women, advocating for change in military policy around sexual abuse, and the importance of using lived experience to both help others and find a sense of closure for themselves.	*There's a big part of me that's really proud that I don't identify in that way, that it's not obvious to people. But I think there's also a big part of me that is, doesn't want to let go of the really wealthy and rich parts that you develop in yourself from being in the military and being a veteran and serving your country. So, I think it's a bit of both and I think it's on a spectrum and I think it changes day to day you know, depending on what's happening and who you're with. (Chrissie, mid-40s, 10+ years)*<br><br>*The people that I am transitioning, I take them to this café around the corner and it's like it's called the Table of Silence, and I tell them, unless you are writing a suicide note out in front of me this will never get back to your Chain of Command, your family, like you can talk to me about anything...Because I still claim them as my soldiers, they are my responsibility...this is why medical health transition is so important...the reason I identify as a veteran because of what I do now as post Defence. (Deirdre, late 30s, 5 years)*<br><br>*So, it's about how you treat the victims afterwards to enable them to become survivors and not leaving them as powerless victims…if that's something that we can achieve, then it means what we've been through actually means something, then it was for a good cause. Then it means that is closure, that gives us closure and that's when you can move on. (Eleanor, 50 years, 20+ years)*
